# Newly implemented community CD4 service in Tshwaragano, Northern Cape province, South Africa, positively impacts result turn-around time

**DOI:** 10.4102/ajlm.v11i1.1376

**Published:** 2022-06-03

**Authors:** Lindi-Marie Coetzee, Naseem Cassim, Deborah K. Glencross

**Affiliations:** 1 National Priority Programme, National Health Laboratory Service, Charlotte Maxeke Johannesburg Academic Hospital, Johannesburg, South Africa; 2 Department of Molecular Medicine and Haematology, University of the Witwatersrand, Johannesburg, South Africa

**Keywords:** CD4 testing, district laboratory, turn-around-time, immunology, HIV, operational science

## Abstract

**Background:**

The Northern Cape is South Africa’s largest province with an HIV prevalence of 7.1% versus a 13.5% national prevalence. CD4 testing is provided at three of five National Health Laboratory Service district laboratories, each covering a 250 km precinct radius. Districts without a local service report prolonged CD4 turn-around times (TAT).

**Objective:**

This study documented the impact of a new CD4 laboratory in Tshwaragano in the remote John Taolo Gaetsewe district of the Northern Cape, South Africa.

**Methods:**

CD4 test volumes and TAT (total, pre-analytical, analytical, and post-analytical) data for the Northern Cape province were extracted for June 2018 to October 2019. The percentage of CD4 results within the stipulated 40-h TAT cut-off and the median and 75th percentiles of all TAT parameters were calculated. Pre-implementation, samples collected at Tshwaragano were referred to Kimberley or Upington, Northern Cape, South Africa.

**Results:**

Pre-implementation, 95.4% of samples at Tshwaragano were referred to Kimberley for CD4 testing (36.3% of Kimberley’s test volumes). Only 7.5% of Tshwaragano’s total samples were referred post-implementation. The Tshwaragano laboratory’s CD4 median total TAT decreased from 24.7 h pre-implementation to 12 h post-implementation (*p* = 0.003), with > 95.0% of results reported within 40 h. The Kimberley laboratory workload decreased by 29.0%, and testing time significantly decreased from 10 h to 4.3 h.

**Conclusion:**

The new Tshwaragano CD4 service significantly decreased local TAT. Upgrading existing community laboratories to include CD4 testing can alleviate provincial service load and improve local access, TAT and efficiency in the centralised reference laboratory.

## Introduction

**FIGURE 1:**
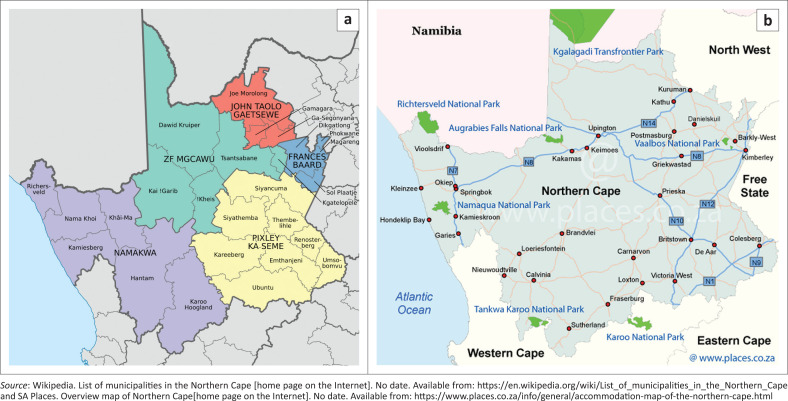
Districts and sub-districts serviced by the community CD4 testing facilities in the Northern Cape province of South Africa (September 2020). (a) major towns (b) major roads between district seats (towns).

The Northern Cape province occupies 30.0% of the land area of South Africa (372 889 km^2^) yet is the most sparsely populated due to the arid climate, with a population of 1.26 million people (3.2 people/km^2^), making up only 2.2% of the national population. The province consists of five districts (the respective main towns where the district seats are situated are indicated in brackets): Namakwa (Springbok), Pixley ka Seme (De Aar), Zwelentlanga Fatman Mgcawu (Upington), Frances Baard (Kimberley), and John Taolo Gaetsewe (Tshwaragano, previously known as Kuruman) (Figure 1^1,2^). Distances between major towns range from 236 km (drive time: 2 h and 24 min) to 775 km (drive time: 7 h and 10 min).

In 2019, the Northern Cape reported an HIV prevalence of 7.1% (i.e. 81 778 people living with HIV), with an estimated 90.0% of patients diagnosed and 83% on anti-retroviral treatment (ART).^3^ This HIV prevalence is significantly lower than the national prevalence of 13.5% and at the lower end of provincial estimates, ranging from 18.2% (2 029 470 people living with HIV) in KwaZulu-Natal to 6.8% (452 210 people living with HIV) in the Western Cape province. The Northern Cape province has the lowest reported density of people living with HIV (0 per 5 km^2^) compared to Gauteng which has over 13 000 people living with HIV per 5 km^2^.^3^ Although it has the most healthcare facilities per 1000 people living with HIV (4.5), more than 10.0% of the population live more than a 2-h drive away from their nearest facility.^1^

In South Africa, a universal ‘test-and-treat’ approach was adopted for the diagnosis and treatment of HIV/AIDS patients.^4,5,6,7,8,9^ Late HIV presentation and a high disease burden^8,9,10,11^ remain challenges in the South African HIV programme. CD4 testing, although no longer required for eligibility for antiretroviral therapy initiation, still plays a valuable role in the identification of HIV-positive patients with advanced disease (CD4 counts < 200 cells/µL) or very advanced disease (CD4 counts < 100 cells/µL).^4,5,6,7,8,10,12,13,14,15,16,17,18,19,20,21^ The local standard of care in South Africa recommends patients returning for care within seven days of HIV diagnosis.^12^ CD4 counts from this visit are used to fast-track patients onto antiretroviral therapy and for the treatment of other HIV-related opportunistic diseases such as tuberculosis and cryptococcal disease.^15,18,22^

In South Africa, CD4 services are offered through the National Health Laboratory Service (NHLS) at 46 testing laboratories placed strategically to service public healthcare facilities consisting of hospitals and primary healthcare clinics in both urban and rural settings. In 2019, 2.8 million CD4 tests were performed across South Africa, with 2.2% done in the Northern Cape province, compared to 34.0% in KwaZulu-Natal and 21.0% in the Gauteng province. The 2020 mid-year population statistics and annual NHLS CD4 testing data (2019) reported that an average of 4.4% of all South Africans received a CD4 test, with 4.7% in the Northern Cape compared to 8.0% per capita in the KwaZulu-Natal province of South Africa.^23^

As only 46 of the 268 NHLS laboratories provide a CD4 testing service, samples are not always tested locally. All samples get registered onto the NHLS laboratory information system (LIS) upon arrival at one of these laboratories, even if CD4 testing is not provided. For this paper, two types of sample referrals are described: one where samples from primary health care facilities and hospitals within respective service precincts are received and registered at smaller, non-testing source laboratories and referred to a CD4 testing laboratory (inter-laboratory referral), and another where samples from hospitals and primary healthcare facilities within the immediate service precinct or catchment area are received, registered and tested at the same laboratory.

Before 2019, three CD4 testing laboratories serviced the entire Northern Cape province, with Kimberley as the local referral testing site for the region (Frances Baard district) receiving samples via inter-laboratory referral or directly from its sister hospitals and other clinics within the direct service precinct. The De Aar CD4 laboratory (Pixley ka Seme district) was implemented in 2012,^24^ and the Upington CD4 laboratory (Zwelentlanga Fatman Mgcawu district) in the following year, after identifying gaps in service by the integrated service delivery model.^25^ CD4 tests from the John Taolo Gaetsewe district (with Tshwaragano as the seat town) were primarily referred to the Kimberley laboratory. The Tshwaragano laboratory is 236 km from Kimberley, 266 km from Upington, and 480 km from the De Aar laboratory (Figure 1) and provides general pathology services to 38 public health facilities within a 200 km service precinct radius.

Laboratory service efficiency and overall performance are typically measured by turn-around time (TAT).^26,27,28,29,30^ In the NHLS, total TAT is reported and includes pre-analytical or laboratory-to-laboratory TAT, that is from first registration onto LIS to sample receipt at the testing laboratory; the analytical registered-to-test TAT component, reporting the time between sample receipt/registration at the testing laboratory to test result entry into the LIS,^28,31^ and the post-analytical TAT, that is the time from result entry at the testing laboratory to authorisation/review by a senior technologist (test-to-review). TAT components that are currently not captured include the time from venepuncture to sample dispatch, travel time to the nearest NHLS laboratory, potential delays at the receiving laboratory before sample registration, and delays of report delivery to the requesting site after the review/release of results. These factors are incorporated in the NHLS CD4 TAT guidelines, requiring that 90% of CD4 results be reported within 40 h of first registration onto the LIS to adhere to the local standard of care (return within seven days).

In April 2018, a routine internal review of annualised provincial TAT revealed that the burden of CD4 testing on the Kimberley laboratory could be alleviated and service delivery improved by further decentralising CD4 testing to Tshwaragano, John Taolo Gaetsewe district (data not published). This study aimed to document the impact of implementing a CD4 testing service in the existing Tshwaragano laboratory on TAT as a measure of laboratory performance. The pre- and post-implementation total and component CD4 testing TAT and the percentage of samples reported within the NHLS-stipulated TAT threshold of 40 h are reported for Tshwaragano and the centralised testing laboratory in Kimberley, Northern Cape, South Africa.

## Methods

### Ethical considerations

Ethics clearance was obtained from the University of the Witwatersrand (M1706108, approved for five years from 13 July 2017). This was a retrospective study using CD4 sample data from the corporate data warehouse, with no patient consent required and no patient identifiers captured.

### Study design

A cross-sectional study design was used to analyse TAT data from CD4 testing laboratories in Kimberley, Upington, De Aar and Tshwaragano, Northern Cape, South Africa. Only data with a reviewed CD4 result between June 2018 to October 2019 were analysed. CD4 testing at all of the Northern Cape laboratories was done using the Beckman Coulter Aquios CL (continuous loader) cytometer system (Beckman Coulter, Miami, Florida, United States).^32^ This system is ideal for low to medium-high test volumes (10–120 samples per day) and is user-independent, with sample preparation and analysis performed in a closed system requiring minimal operator hands-on time, an especially useful feature for sites with limited dedicated/skilled staff. The Aquios CL platform performance correlated well with previously used systems such as the COULTER EPICS XL-MCL™^33^ and FC500 multi-plate loader (MPL)^34^ as per validations done before the national roll-out^35,36^ and instrument verifications done per testing site as part of the implementation process.^37,38,39^

### Data preparation

Data were extracted from the Central Data Warehouse of the NHLS as password-protected files. The extracted data included the following variables: episode number, referral laboratory, testing laboratory, facility name, province, health district, sub-district, date first registered on the LIS, date tested, date reviewed (result released), total TAT, laboratory-to-laboratory TAT, registered-to-test TAT, and test-to-review TAT. Data were categorised as ‘Not referred’ where the referral and testing laboratory was the same and as ‘Referred’ where the testing and referral laboratories differed. Data with invalid dates and a total TAT > 120 h or < 1 h were excluded. A total TAT of 1 h is set as the minimum analytical acquisition and reporting time on the Aquios platform, while a total TAT > 120 represented the upper limit of the CD4 testing window (five days), beyond which a specimen is no longer suitable for analysis.^40,41^ Data were categorised to distinguish between the pre-implementation (June 2018 to October 2018) and post-implementation (November 2018 to October 2019) periods; the month of implementation (October 2018) was categorised as pre-implementation as samples were still being referred to Kimberley.

### Implementation procedures at the Tshwaragano laboratory

**FIGURE 2:**
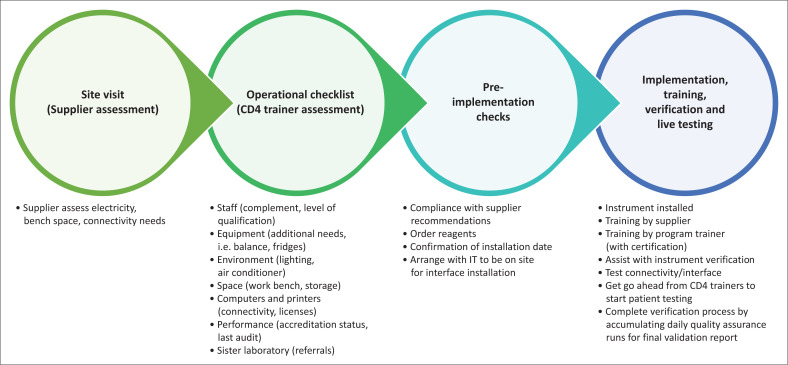
Summary of the standardised process for establishing new CD4 testing facilities in the National Health Laboratory Service, South Africa, September 2020.

All of the procedures undertaken to ensure that the Tshwaragano laboratory was ready to offer quality CD4 testing were documented. Typically, a site inspection was done by the service provider and an on-site operational checklist was completed by a CD4 trainer to assess requirements for implementation. Several readiness checks were confirmed before the commencement of instrument implementation, training, verification, and reportable patient testing (Figure 2).

### Satisfaction questionnaire

A Likert scale satisfaction survey was circulated to the 15 health facilities with the highest test volumes referred to Tshwaragano laboratory for CD4 testing. On a scale from ‘1’ (poor) to ‘5’ (very good), the facility manager/senior nurse at these clinics rated the overall service rating, average TAT and CD4 test result delivery of the CD4 service pre-and post-implementation of local CD4 testing at the Tshwaragano laboratory.

### Data analysis and statistics

Microsoft Excel (Redmond, Washington, United States) and SAS^®^ statistical software 9.4 (Cary, North Carolina, United States) were used to analyse the data. Graphs were created with GraphPad Prism software, version 7.0 for Windows (GraphPad Software, San Diego, California, United States).

Pre-implementation of the CD4 testing laboratory at Tshwaragano, the overall and monthly number of referred samples tested by the three then active CD4 laboratories in the region (Kimberley, De Aar and Upington) were assessed. TAT parameters to determine laboratory performance/efficiency included the total TAT, the interquartile range (IQR) of the total TAT, the percentage of all tested samples within the NHLS-stipulated 40-h TAT cut-off, and the median TAT for the three TAT components. Due to the non-Gaussian distribution of TAT data,^28^ the median and 75th percentile of total TAT was calculated and reported for this study; this was to allow the analysis of the majority of samples without the influence of outliers or, in Gaussian terms, the long right tail, that is CD4 samples with a total TAT exceeding 120 h (typically outside the 99th percentile). The monthly median and 75th percentile total TAT for the Tshwaragano and Kimberley laboratories were reported.

Pre- and post-implementation test volumes and TAT parameters (total TAT and TAT components) were compared at the provincial level (all Northern Cape results) and individual laboratory level using either the unpaired, non-parametric Mann-Whitney analysis for two groups or the unpaired, non-parametric Kruskal-Wallis test for three or more groups. *P*-values of < 0.01 were considered statistically significant and *p* < 0.001 highly significant.

## Results

Exclusion criteria identified 10 131 samples (1.5%) with a total TAT exceeding 120 h and two samples with a total TAT of less than 1 h. After applying the exclusion criteria, CD4 data for 83 232 samples collected during the study period were analysed. Of all CD4 samples tested in the Northern Cape province, the median CD4 count was 437 cells/µL (IQR: 237–658 cells/µL), the median total TAT was 19.8 h (IQR: 11.9–31.9 h), and 96% of all tested samples were within the NHLS-stipulated 40-h TAT cut-off.

### Pre- and post-implementation CD4 test volumes

**FIGURE 3:**
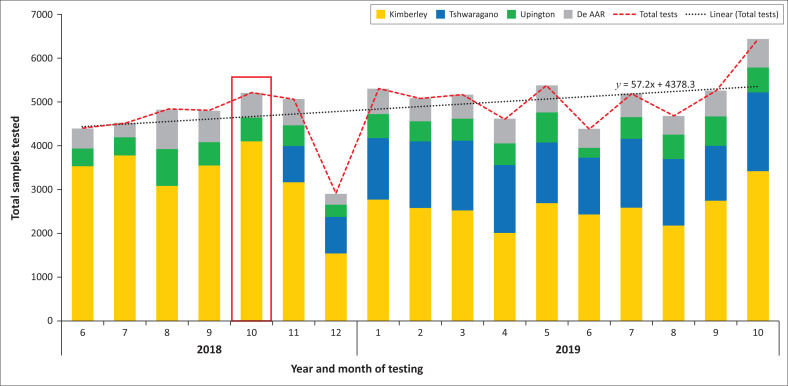
Total CD4 test volumes of laboratories in the Northern Cape, South Africa, June 2018 to October 2019. The red rectangle indicates the implementation month of October 2018. The red dotted line shows total test volumes per month, with the linear trend line (black dotted line) and equation indicated.

During the pre-implementation and implementation months (5 months), 23 754 CD4 tests were performed across the province: 75.9% (*n* = 18 030) at the Kimberley laboratory, 12.6% (*n* = 2988) at De Aar, and 11.5% (*n* = 2736) at the Upington laboratory (Figure 3). There were 13 714 samples registered at the Tshwaragano laboratory during the study period, of which 95.4% were referred to and tested at the Kimberley laboratory, and 4.6% were referred to and tested at the Upington laboratory. The 12-month follow-up post-implementation period reported 59 478 samples tested across the four laboratories. Of these, 42 879 (72.1%) were tested at the existing laboratories: Kimberley (*n* = 30 575; 51.4%), De Aar (*n* = 6356; 10.7%) and Upington (*n* = 5948; 10.0%). The newly implemented Tshwaragano CD4 laboratory tested 27.9% (16 599) of the total Northern Cape CD4 samples over the 12-month post-implementation period, becoming the second-largest testing site in the Northern Cape. Overall, a slight increase in total provincial CD4 test numbers of 4% per month was noted post-implementation (Figure 3).

### Referral patterns during the pre- and post-implementation periods

During the pre-implementation phase, 32.9% (7829/23 754) of all CD4 tests done in the Northern Cape province were referred from source laboratories (where samples were first registered on the NHLS LIS), while the remaining tests (*n* = 15 925; 67.0%) were registered and tested at the same laboratory. At the Kimberley laboratory, 62.7% (*n* = 11 296) of samples tested were from local hospitals and health facilities. Referred source laboratory samples tested at this laboratory came from the Tshwaragano (36.3%; 6541), Upington (0.7%; 131) and De Aar (0.3%; 57) laboratories (Table 1). Five samples (0.02%) were referred from outside of the Northern Cape provincial borders. The De Aar and Upington laboratories tested most of the samples from their local precincts, contributing 75.8% (*n* = 2266 of 2988; De Aar) and 86.4% (*n* = 2363 of 2736; Upington) of their workloads. Samples were only referred to Kimberley by these laboratories due to local instrument or network downtime.

**TABLE 1:** CD4 testing workload at the four precinct laboratories in the Northern Cape province, South Africa, during the pre-implementation and post-implementation of the Tshwaragano CD4 laboratory between June 2018 and October 2019.

Testing laboratory	Referral laboratory	% of total samples tested (CD4 testing workload)			
		Pre-implementation		Post-implementation	
		Local precinct workload	Referred workload	Local precinct workload	Referred workload
**Kimberley**	Sourced and tested locally	62.7	-	92.2	-
	Referred from Tshwaragano	-	36.30	-	4.10
	Referred from De Aar	-	0.70	-	1.60
	Referred from Upington	-	0.30	-	2.10
	Referred from Joe Morolong‡	-	0.02	-	0.03
**De Aar**	Sourced and tested locally	75.8	-	92.5	-
	Referred to Kimberley	-	24.20	-	7.50
**Upington**	Sourced and tested locally	86.4	-	87.7	-
	Referred from Tshwaragano	-	11.60	-	1.40
	Referred to Kimberley	-	2.10	-	10.90
**Tshwaragano†**	Sourced and tested locally	0.0	-	92.0	-
	Referred to Kimberley	-	95.40	-	7.50
	Referred to Upington	-	4.60	-	0.50

† , The newly-established CD4 testing laboratory;

‡ , This laboratory is in the North West province that borders the Northern Cape.

Post-implementation, 91.97% of all CD4 test samples received at the Tshwaragano laboratory (*n* = 16 599) were tested locally (*n* = 15 266), with 1250 samples referred to the Kimberley laboratory (7.5%) and 83 samples referred to the Upington laboratory for testing (0.5%). For the same period, the Kimberley CD4 workload was made up almost entirely of locally received samples (92.2%) (Table 1); only 7.8% of their total CD4 test volumes (*n* = 2385 samples of 30 575) were received from referring laboratories, that is Tshwaragano (4.1%), Upington (2.1%) and De Aar (1.6%). The proportion of local samples tested at the Kimberley laboratory thus increased by 29.5% between the pre- and post-implementation periods, while the average number of tests performed monthly decreased from 3606 pre-installation to 2547 per month post-installation (29.4%).

The overall median CD4 count pre-implementation was 442 cells/µL: 397 cells/µL in Upington, 419 cells/µL in Kimberley and 451 cells/µL at the De Aar laboratory. The differences in median CD4 count between the laboratories were not clinically significant. The post-implementation overall median CD4 count was 436 cells/µL: 406 cells/µL in Upington, 440 cells/µL in Kimberley and Tshwaragano, and 427 cells/µL at the De Aar laboratory. Differences in median CD4 count pre- versus post-implementation for the Northern Cape province were not statistically significant (Kruskal-Wallis, *p* = 0.139).

### Turn-around time: Pre- versus post-implementation

The median total TAT across the four Northern Cape province testing laboratories during the whole testing period was 19.8 h (IQR: 11.9–31.9 h). The pre-implementation phase median total TAT for the three laboratories was 21.65 h (IQR: 16.7–34.1 h) compared to 18.5 h (IQR: 10.9–31.4 h) in the post-implementation phase across four laboratories (Mann-Whitney *p* = 0.0001, highly significant).

**FIGURE 4:**
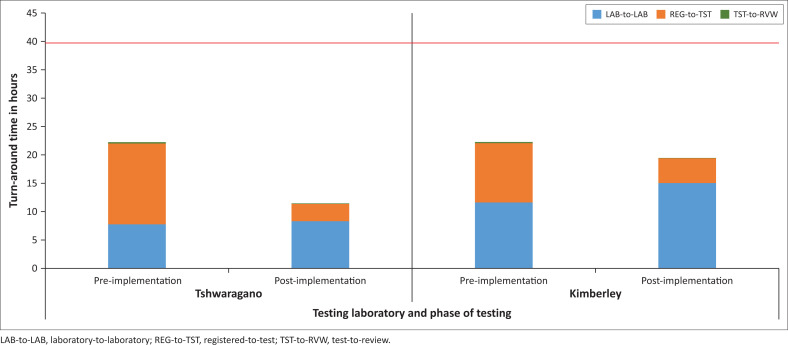
CD4 testing turn-around time components at the Tshwaragano and Kimberley laboratories in the Northern Cape, South Africa during the pre- and post-implementation months (June 2018 to October 2019). LAB-to-LAB: time from first registration on the laboratory information system to sample receipt in the testing laboratory; REG-to-TST: time from registration/receipt at the testing laboratory to test result entry on the laboratory information system; TST-to-RVW: time from test result entry to verification by a senior staff member and release to requesting clinic/physician.

**FIGURE 5:**
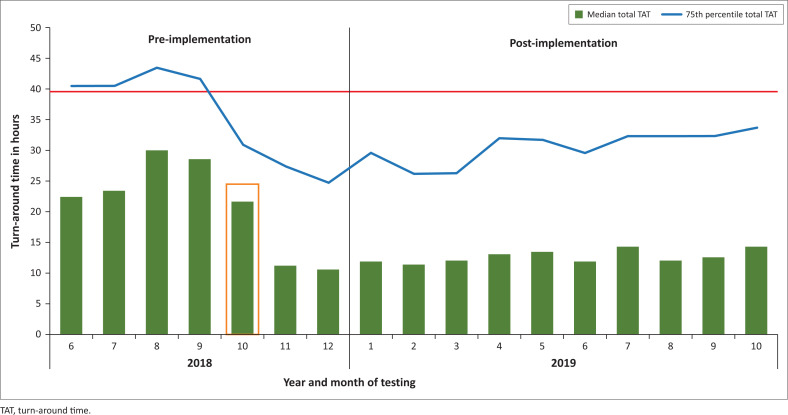
CD4 testing median total turn-around times at the Tshwaragano laboratory in the Northern Cape province of South Africa from June 2018 to October 2019. The orange rectangle indicates the month of CD4 implementation at the Tshwaragano laboratory and the solid red line shows the organisational CD4 cut-off of 40 h.

For the Tshwaragano laboratory, there was a significant decrease in median total TAT from 24.7 to 12 h pre- versus post-implementation (Mann-Whitney *p* = 0.0009), mainly due to a sharp decline in the median testing time (registered-to-test) TAT from 14 h to 3 h (Figure 4). Comparatively, the median total TAT at the Kimberley laboratory only decreased from 23.0 h to 22.2 h (not significant), although their analytical phase (registered-to-test) TAT significantly decreased from 10 h to 4.3 h. For the new Tshwaragano laboratory, 75th percentile total TAT values were monitored monthly (Figure 5) and observed to decrease significantly to below the stipulated cut-off of 40 h, stabilising around 32 h after July 2019. The proportion of samples reported within the stipulated 40-h threshold at Tshwaragano changed significantly from 84.0% (pre-implementation) to 92.5% post-implementation. Since November 2019, the efficiency of the Tshwaragano laboratory has improved, with > 95.0% of tested samples within the NHLS-stipulated 40-h TAT threshold month-on-month (as per ongoing monthly TAT dashboard reports). The Kimberley, De Aar and Upington CD4 laboratories were able to sustain a 75th percentile total TAT of < 40 h, with > 90.0% of samples meeting the < 40-h cut-off monthly after the reviewed period.

The test-to-review TAT component did not impact the total TAT as all laboratories reported TAT times < 1 h (median: 10 min).

### Questionnaire results

The pre-implementation assessment score was 3 (performance rated as average), with delayed result delivery being the most common reason for the rating provided. The post-implementation score was 5 (very good), with faster result delivery (most within 24 h) being the most cited improvement. Respondents observed that patients returned earlier than the stipulated seven days to collect their results (within 2–4 days). Nonetheless, feedback received highlighted ongoing challenges of poor connectivity that affected access to results on the Internet-based LIS and delivery of SMS printer results in remote outlying facilities.

## Discussion

This paper describes the positive impact of the implementation of a new CD4 testing facility in the Tshwaragano laboratory of the Northern Cape, South Africa, and the subsequent improved CD4 testing TAT in this region. The implementation of the Tshwaragano laboratory positively impacted the region’s referral laboratory at Kimberley, where average monthly workload volumes decreased by 29.4 %, enabling a better workflow and more efficient service delivery. Post-implementation of CD4 testing services at the Tshwaragano laboratory, all the testing sites, that is the three community CD4 laboratories at De Aar, Upington and Tshwaragano, and the referral laboratory at Kimberley, could focus solely on managing their local precinct workload, with > 85.0% of samples registered and tested on-site per laboratory. Expectedly, no significant changes were noted in the median CD4 count overall and per laboratory as the patient base accessing care remained unchanged.

The most dramatic impact was seen in the testing phase (registered-to-test) TAT, which decreased significantly at both the Tshwaragano (14 h to 3 h) and Kimberley (10 h to 4 h) laboratories, contributing to the overall decrease in median total TAT at these laboratories. This could be attributed to a better-organised workflow, where all samples are collected, registered and available in the laboratory by the start of the testing shift (night). This is especially relevant as referred samples often get set aside and tested only after local sample testing is done. The average daily test volumes for the Tshwaragano and Kimberley laboratories (< 160 samples per day) are well within the capacity of the testing platform (Aquios) and the number of instruments per laboratory, resulting in better instrument utilisation.

The test-to-review TAT component at all testing laboratories was < 1 h and did not impact total TATs, that is, there were no delays associated with waiting for qualified staff members to review results. Further streamlining of this process was made possible by the introduction of auto-review in March 2020.^42^

There was a slight increase (~4%) in the monthly average CD4 tests conducted in the Northern Cape province during the pre-and post-implementation periods. The latter could be attributed to the availability of local testing at Tshwaragano and the addition of six additional referring clinics (personal communication with laboratory management) since the commencement of CD4 services.

The outcomes of the satisfaction rating questionnaire were a testament to the success of this implementation, with an overall post-implementation rating of 5 (vs 3 pre-implementation). Improved local CD4 report delivery (due to shortened local TAT) could see patients return for their results earlier than the stipulated seven days. This is especially relevant in patients with severe HIV disease (CD4 < 100 cells/µL) to enable the fast-tracking of serum cryptococcal antigen testing and rapid initiation of treatment in cryptococcal antigen-positive cases. As monitored through the NHLS weekly/monthly TAT dashboard reports, the Tshwaragano laboratory maintained a median total TAT and 75th percentile total TAT of < 20 h for the last financial year (April 2020 to March 2021), with > 98% of samples tested within the 40-h cut-off.

This study supports the findings of previous studies^24,28,31,43^ and demonstrates how decentralising CD4 testing services to district or community facilities according to a tiered service delivery model^25^ can improve the TAT at the decentralised site. Smaller community laboratories with existing laboratory infrastructure offering more generalised pathology services can reliably implement CD4 services, effectively eliminating delays due to the transport of samples historically sent to centralised testing facilities. NHLS community laboratories are usually located in the seat of the respective districts and generally serve large service precincts, typically supporting up to 40 or more health facilities. With minimal training effort and cost^25,43^ (benchtop installation, installation of LIS interface with instruments, staff training, and testing platforms on national service level agreement), laboratories can be upgraded to include CD4 testing in their repertoire of services. Decentralised services also improve coordinated technical efforts across the parent province (the Northern Cape in this instance), where CD4 laboratories can, in line with the integrated service delivery model service plan,^25^ operate as a consolidated network with two-way support between the main reference laboratory (in Kimberley) and sister community laboratories, thereby ensuring uninterrupted self-sustaining service.

### Limitations

The reported TAT only reflects laboratory TAT and not the full sample journey from a patient to a result-in-hand, which may impact patient management (i.e., leading to patients returning for results, loss to follow-up, and/or delays in the initiation of therapy). However, this paper aimed to show the impact on TAT at the laboratory level and did not assess the impact on patient management.

### Conclusion

Using existing infrastructure, upgrading an existing district NHLS laboratory to include CD4 testing is possible with minimal intervention. District CD4 testing laboratories can service their local health clinics and hospitals without delays due to sample referral, reducing the total TAT and allowing earlier patient support/intervention. The implementation of a fourth district CD4 testing facility in the Northern Cape province resulted in the local testing of > 90% of samples from their catchment areas while maintaining excellent performance.
